# Combining chondroitinase ABC and growth factors promotes the integration of murine retinal progenitor cells transplanted into *Rho*^−/−^ mice

**Published:** 2011-06-29

**Authors:** Jian Ma, Mostafa Kabiel, Budd A. Tucker, Jian Ge, Michael J. Young

**Affiliations:** 1 State Key Laboratory of Ophthalmology, Zhongshan Ophthalmic Center, Sun Yat-sen University, Guangzhou, China; 2 Department of Ophthalmology, Schepens Eye Research Institute, Harvard Medical School, Boston, MA; 3 Department of Ophthalmology, Mansoura University, Mansoura, Egypt

## Abstract

**Purpose:**

The aim of this study is to investigate the synergistic effect of chondroitinase ABC and growth factors in the integration of murine retinal progenitor cells (mRPCs) transplanted into *Rho*^−/−^ mice.

**Methods:**

mRPCs from P1 green fluorescent protein-transgenic mice were isolated and expanded for transplantation. All mRPCs of 20 passages or less were transplanted into the subretinal space of B6 mice together with chondroitinase ABC, and into Rho^−/−^ mice combined with chondroitinase ABC, N-[N-(3, 5-Difluorophenacetyl)-L-alanyl]-S-phenylglycine t-butyl ester (DAPT), and insulin growth factor (IGF)-1. Cell counts were used to examine the migration and survival rate of mRPCs in B6 mice. Immunohistochemistry was used to evaluate the differentiation and integration of mRPCs in B6 and Rho^−/−^ mice.

**Results:**

Our results show that substantial numbers of mRPCs migrated and survived in the retina when transplanted with chondroitinase ABC into B6 and *Rho*^−/−^ mice. Chondroitinase ABC disrupted the glial scar around the mRPCs in the subretinal space. Only a few mRPCs expressed recoverin in B6 mice. More mRPCs expressed rhodopsin, recoverin, and synaptophysin after transplantation into *Rho*^−/−^ mice when combined with chondroitinase ABC and growth factors.

**Conclusions:**

The synergistic effect of chondroitinase ABC and growth factors facilitates the anatomic integration of mRPCs transplanted into *Rho*^−/−^ mice.

## Introduction

Cell-replacement therapy is a novel strategy used to treat irreversible retinal neurodegeneration diseases, such as age-related macular degeneration (AMD) and retinitis pigmentosa (RP), which are characterized by photoreceptor cell death [1]. A range of studies suggests that the use of stem cells to achieve this goal is feasible. So far, multiple cell types have been used for research including neural progenitor cells [2], hippocampal progenitor cells [3], bone marrow-derived cells [4], retinal progenitors cells (RPCs) [5], postmitotic photoreceptor precursor cells [6], retinally-directed human ES cells [7], and photoreceptors derived from human induced pluripotent stem cells [8]. All of these cells have been shown to survive in the recipient for a few weeks and many can even restore some visual function. Among these studies, several types of recipients have been used for transplantation, including *Rho*^−/−^ mice [5,9], laser-injury mice [10,11], rd mice, a model of retinitis pigmentosa [12], and *Crx*-deficient mice [7].

Though recent studies show some functional restoration after subretinal cell transplantation [5-7], the low efficiency of integration remains a problem for cell-based transplantation. Only 0.01% of transplanted cells survive and even fewer integrate with the host retina [6]. To replace the photoreceptor cells, transplanted cells must migrate and integrate into the degenerated outer nuclear layers (ONL).

Physical barriers (outer limit membrane, OLM), glial scarring, and immune rejection are three major obstacles for the migration of donor cells [1,13-15]. In addition, activated Müller cells and microglia are thought to increase extracellular matrix (ECM) components such as chondroitin sulfate proteoglycans (CSPGs), which have been shown to limit axon extension in the brain [16-20]. Also, the presence of a glial boundary and two inhibitory molecules including CD44 and neurocan, accumulated at the abutting retina’s interface, seemed to correlate with a lack of integration. Interestingly, the migration of cells was accompanied by increased glial reactivity within the abutting retina. Migration may relate to matrix metalloproteinase 2 (MMP2) production by the host glial cells, which can specifically degrade both neurocan and CD44 [14,21]. Similarly, CSPGs play a vital role in preventing the migration and integration of grafted cells into degenerating retina [22]. Previously, several methods were employed to facilitate the movement of donor cells, including using glial fibruillary acidic protein (GFAP)/Vimentin knockout mice [23], progenitor cell/biodegradable MMP2-PLGA polymer [24], α-Aminoadipate (AAA) [25,26], and chondroitinase ABC [22,27,28]. Chondroitinase can promote functional recovery in the damaged CNS via disruption of extracellular matrix components [29]. It has also been shown to promote functional axonal regeneration, functional repair, and plasticity of the chronically injured spinal cord [30,31]. Chondroitinase ABC enhances synaptogenesis between transplanted and host neurons in the chemical-induced model of retinal degeneration [28]. However, there is no evidence that chondroitinase can induce differentiation. Several growth factors are supposed to promote photoreceptor cell differentiation including N-[N-(3, 5-Difluorophenacetyl) -L-alanyl] -S-phenylglycine t-butyl ester (DAPT) and insulin growth factor (IGF)-1 [32,33]. Although great progress has been made in treating central nervous system (CNS) diseases via the transplantation of stem cells combining chondroitinase and growth factors, the effect of combining chondroitinase and growth factors in eye disease has never been investigated. In this study, we examined the synergic effects of chondroitinase ABC, DAPT, and IGF-1 on the differentiation and integration of mRPCs into *Rho*^−/−^ mice.

## Methods

### Animals

All experiments were performed in compliance with the ARVO Statement for the Use of Animals in Ophthalmic and Vision Research, and all experimental protocols were approved by the Animal Care and Use Committee of the Schepens Eye Research Institute. Green fluorescent protein positive (GFP+) C57BL/6J (B6) mice, C57BL/6J mice (Jackson Laboratory, Bar Harbor, ME) and Rhodoposin null C57B16 mice (*Rho*^−/−^, Peter Humphries, Trinity College, Dublin, Ireland) were housed in a 12 h:12 h light-dark cycle with water and food provided ad libitum.

### Cell isolation and culture

Retinal progenitor cells harvested from the neural retinas of P1 enhanced green fluorescent protein mice were isolated and maintained in culture as previously described [5]. Briefly, neural retinas were dissected from surrounding tissues. Pooled neural retinal tissue was minced and dissociated enzymatically (0.1% collagenase in HBSS; Sigma, St. Louis, MO) at room temperature. Liberated cells were collected through a 100 um mesh strainer, centrifuged, and then resuspended in a neurobasal medium, which was supplemented with epidermal growth factor (EGF; 20 ng/ml), L-glutamine (2 mM; Sigma), nystatin (2,000 U; Sigma), penicillin/streptomycin (100 μg/ml; Sigma), and 2% B-27 supplement and N-2 supplement (Invitrogen, Carlsbad, CA). Subsequently, cells were fed by 50% medium exchange every 2–3 days and were passaged at confluence. Although capable of expansion through more than 60 passages, cells of 20 passages or less were used for transplantation.

### Subretinal transplantation

Mice were placed under general anesthesia with an intraperitoneal injection of ketamine (120 mg/kg) and xylazine (20 mg/kg), followed by local anesthetization with 0.5% proparacaine (Accutome, Malvern, PA) and pupil dilation with 1% tropicamide (Akorn, Lake Forest, IL). The temperature of the mice was maintained at 37 °C during surgery using a heating pad and heat lamp. Transplantation was performed under an operating microscope, as has been described [5,34]. The tip of a 30-gauge needle (BD) was inserted through the sclera into the intravitreal space to reduce intraocular pressure and was then withdrawn. One μl HBSS containing RPC (approximately 100,000 cells), or combined with chondroitinase ABC (0.01 U/μl), or in a cocktail (chondroitianse ABC 0.01 U/μl, DAPT 10 μM, IGF-1 10 μg/ml) was inserted tangentially through the sclera into the sub-retinal space. The injection, using a glass microneedle attached to a 50 μl syringe (Hamilton, Reno, NV) via polyethylene tubing, proceeded slowly to produce retinal detachment in the superior hemisphere around the injection site. There were 9 mice in each group. The age of the mice for transplantation was about 6–8 weeks. Following transplantation, mice were allowed to recover, were returned to their normal housing, and were left for 1 or 3 weeks post transplantation.

### Tissue preparation and histology

The B6 mice in receipt of mRPCs transplants were euthanized at 1 week or 3 weeks after transplantation. The *Rho*^−/−^ mice were euthanized 3 weeks after transplantation. The eyes were fixed in 4% paraformaldehyde, cryoprotected in 10% and 30% sucrose in a 0.1% phosphate buffer, and were sectioned at 8 μm on a cryostat. Tissue sections were immunostained for chondroitin (a marker for NG2 Chondroitin Sulfate Proteoglycan; 1:100; Chemicon, Temecula, CA), glial fibrillary acidic protein (GFAP; a marker for Müller cells; 1:250; Chemicon), recoverin (1:1,000, a marker for rod and cone photoreceptors and some cone bipolar cells; Chemicon), rhodopsin (rod photoreceptors marker; 1:100; Chemicon), protein kinase alpha (PKCα; a marker for bipolar cells; 1:100; Santa Cruz, Santa Cruz, CA), and synaptophysin (a marker for integral membrane protein of small synaptic vesicles in brain and endocrine cells; 1:100; Dako, Carpinteria, CA), followed by reaction with Cy3-conjugated or Cy5-conjugated secondary antibody. Sections were examined with conventional and confocal microscopy.

### Migration and survival evaluation

To evaluate the migration of mRPCs, we calculated the distance that the cells migrated into the retinal layers from the injection site. To quantify the migrated cells’ survival, we counted the mRPCs in the retinal layers, excluding the donor cells in the subretinal space. Cells were counted within one random microscopy field at 40× (n=9).

### Electroretinography recordings

The electroretinograms (ERGs) of *Rho*^−/−^ mice were assessed using a UTAS-E3000 recording system (LKC Technologies, Gaithersburg, MD). Mice were dark-adapted overnight and anesthetized with a mixture of ketamine/xylazine (120 mg/kg and 20 mg/kg, respectively). After pupil dilatation with 1% tropicamide and 1.5% cyclopentolate, each mouse was placed in front of a Ganzfeld bowl (UTAS3000; LKC Technologies). The active electrode (a gold wire loop) was placed on the cornea, the reference electrode was placed on the head, and the ground electrode was placed on the back. ERG responses to a series of increasing-intensity light flashes: 0, 10, and 20 dB were averaged over 10 separate flashes per light intensity. The a-wave amplitude was measured from the baseline to the trough of the first negative wave; the b-wave amplitude was measured from the trough of the a-wave to the peak of the first positive wave or, if the a-wave was absent, from the baseline to the peak of the first positive wave.

### Statistics

Data were represented as mean±SD. Statistical analysis of the data was performed using SPSS13.0, IBM SPSS Data Collection, Chicago, IL; significant differences were examined with the Mann–Whitney U test. p<0.05 was considered significant.

## Results

**Figure 1:**
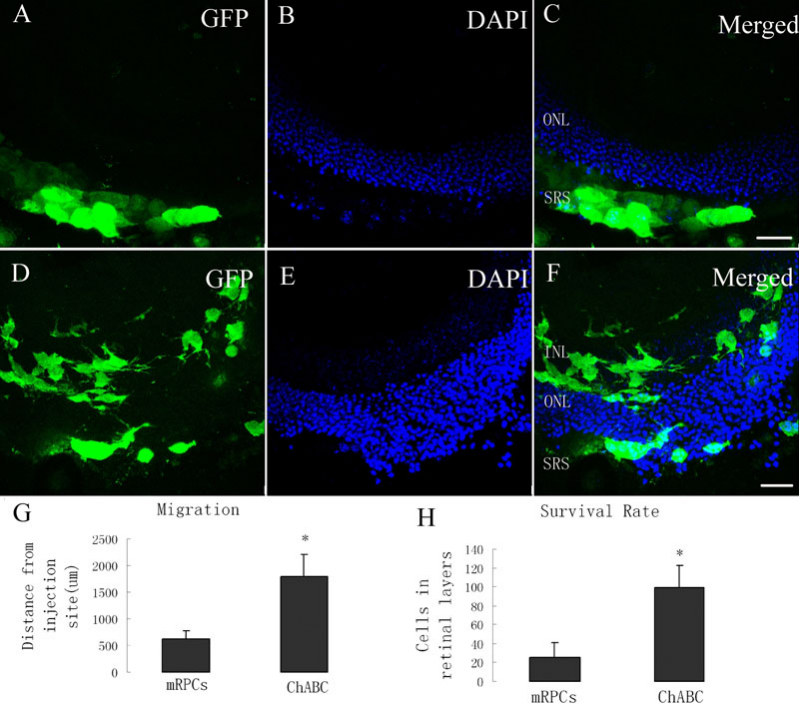
Chondroitinase ABC promotes the migration of murine retinal progenior cells (mRPCs) into B6 mice. **A-C**: One week after transplantation of mRPCs. **D-F**: One week after transplantation of mRPCs combined with Chondroitinase ABC. Murine retinal progenitor cells gather in the subretinal space. Abundant of cells migrated into the retinal layers were seen in **F**. Quantification of the difference of migration distance from the injection site (**G**) and the cells in the retinal layers (**H**). The cells in the chondroitinase ABC treated group have a higher ability to migrate and survive in the retinal layers. Scale bar: 25 μm (**C, F**). **G**, **H**: The columns are mean values, error bars are SD, *p<0.05 (n=9). Abbreviations: inner nuclear layer (INL); outer nuclear layer ONL; and subretinal space (SRS).

To evaluate the effect of chondroitinase ABC on the migration of donor cells, we transplanted the mRPCs combined with chondroitinase ABC into the subretinal space of B6 mice. The effective concentration of chondroitinase ABC was 0.01 U/μl, as shown in a previous study [27]. Murine retinal progenitor cells remained in the subretinal space and seldom migrated into the retinal layers one week after transplantation in the control group (Figure 1C). Substantial migration of mRPCs was seen when the transplant was combined with chondroitinase ABC (Figure 1F). The cells had a higher ability to migrate and survive in the retinal layers in the chondroitinase ABC treated group (Figure 1G,H). The average distance migrated from the injection site in both the control and the chondroitinase ABC treated group was 620.56±159.49 μm and 1795.56±414.76 μm, respectively. The farthest distance migrated from the injection site was 2284 μm in the Chondroitinase ABC treated group. The number of cells in the retinal layers of the control group and the chondroitinase ABC treated group was 25.56±15.63 and 99.22±23.51, respectively, (n=9).

**Figure 2:**
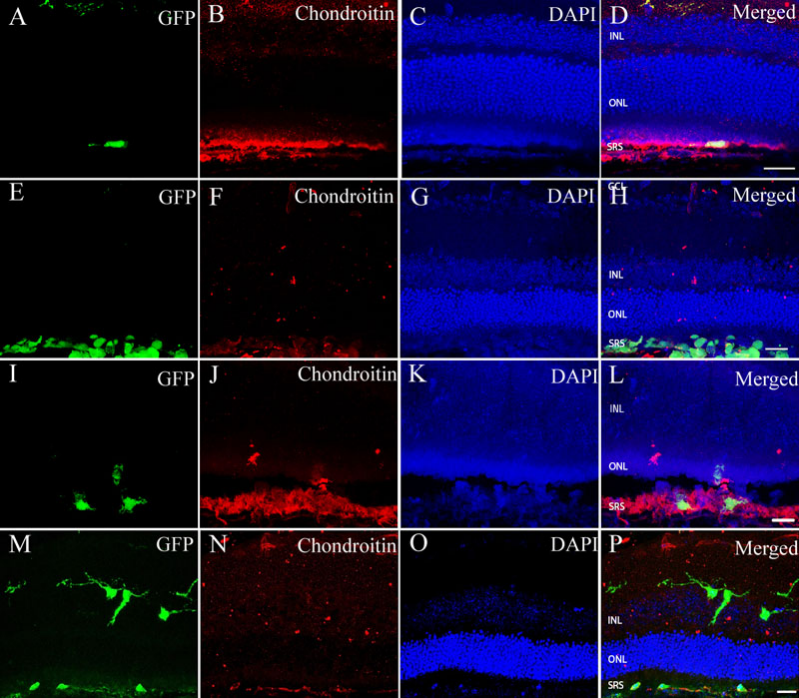
Chondroitinase ABC disrupts the chondroitin sulfate proteoglycans (CSPGs) and glial scar barrier in B6 mice. **A-D**: One week after subretinal transplantation of murine retinal progenior cells (mRPCs). **E-H**: One week after subretinal transplantation of mRPCs combined with Chondroitinase ABC. **I-L**: Three weeks after subretinal transplantation of mRPCs. **M-P**: Three weeks after subretinal transplantation of mRPCs with chondroitinase ABC. Following transplantation of mRPCs and Chondroitinase ABC, the expression of chondroitin (**F**, **N**) is much lower than that of **B** and **J**. Scale bar: 25 μm (**D**, **H**), 20 μm (**L**, **P**). Abbreviations: GCL, ganglion cell layer (GCL); inner nuclear layer (INL); outer nuclear layer (ONL); and subretinal space (SRS).

As shown before, chondroitin sulfate proteoglycans (CSPG) plays an important role in preventing the migration and integration of donor cells [22]. One week after transplantation, chondroitin was upregulated in the outer nuclear layer and in the vicinity of the donor cells (Figure 2D). Comparably, the chondroitin expression around mRPCs was much lower in the chondroitinase ABC treated group (Figure 2H). Also, there was no chondroitin located in the outer nuclear layers. Three weeks after transplantation, chondroitin was still evident in the outer nuclear layer and in the vicinity of the cells that resided in the subretinal space (Figure 2L), albeit with faint expression. Some donor cells extended their dendrites in the chondroitinase ABC treated group (Figure 2P).

**Figure 3:**
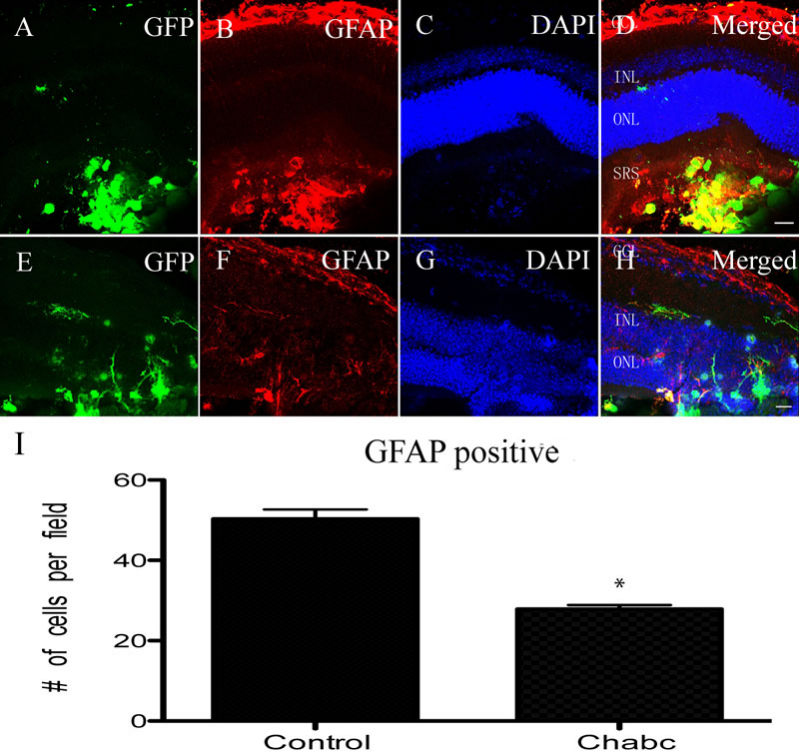
Glial fibrillary acidic protein (GFAP) expression of murine retinal progenior cells (mRPCs) in B6 mice. **A-D**: Three weeks after transplantation of mRPCs. **E-H**: Three weeks after transplantation of mRPCs combined with Chondroitinase ABC. **I**: The average number of GFAP-positive cells was counted per microscopic field. The columns are mean values, error bars are SD, *p<0.05 (n=9). The cells gathered in the subretinal space almost express GFAP. Following transplantation of mRPCs and Chondroitinase ABC, the cells migrated to the retinal layers and seldom express GFAP. Scale bar: 20 μm (**D**, **H**). Abbreviations: ganglion cell layer (GCL); inner nuclear layer (INL); outer nuclear layer (ONL); and subretinal space (SRS).

The mRPCs were most often seen in the subretinal space and most expressed GFAP in the control group. Less cells migrated into the retinal layers (Figure 3D). In the chondroitinase ABC group, the cells migrated into the retinal layers and extended their process to the inner nuclear layer and inner plexiform layer (Figure 3H). Only a few cells expressed GFAP. GFAP was still seen in the inner nuclear layer, retinal ganglion layer, and in the donor cells located in the subretinal space (Figure 3D,H). Compared to the control group, the cells had a higher ability to migrate and a lower incidence of differentiation into glial cells.

**Figure 4:**
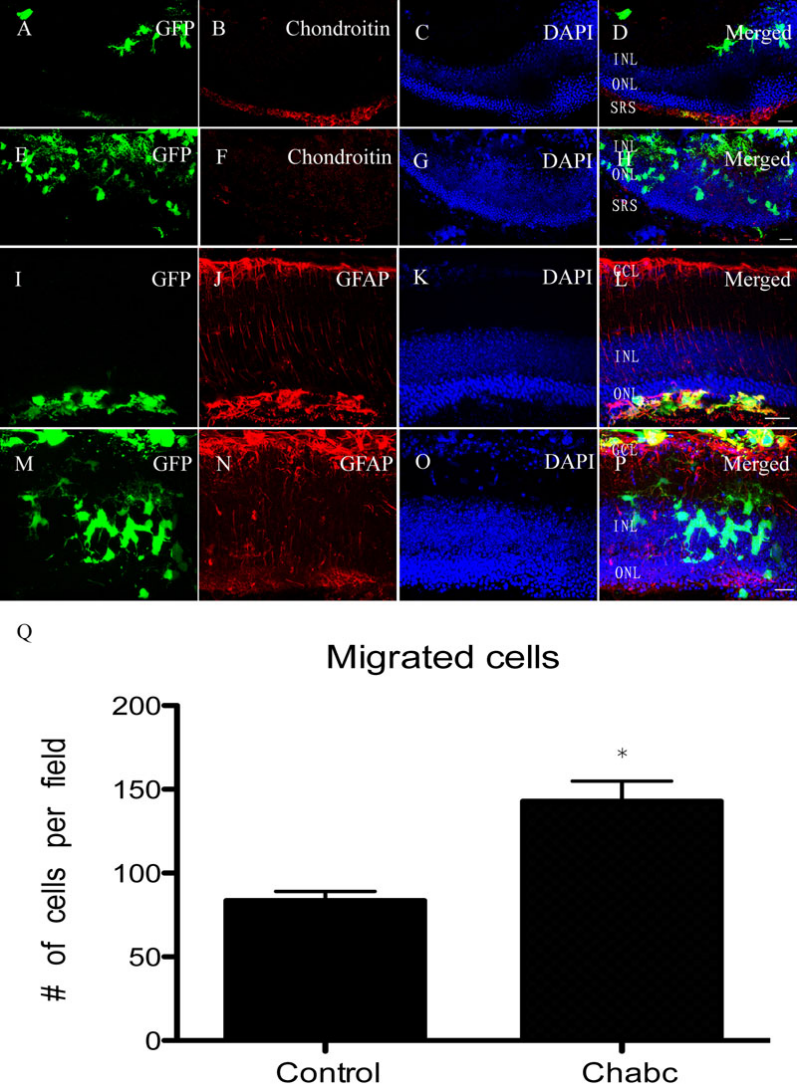
Three weeks post transplantation of murine retinal progenior cells (mRPCs) into *Rho*^−/−^ mice. The first row (**A**, **B**, **C**, **D**) and the third row (**I**, **J**, **K**, **L**) were mRPCs groups as control. The second row (**E**, **F**, **G**, **H**) and the fourth row (**M**, **N**, **O**, **P**) were chondroitinase ABC treated groups. **Q**: The average number of cells in the retinal layers was counted per microscopic field. The columns are mean values, error bars are SD *p<0.01 (n=9). The chondroitin expression in **F** was lower than that in **B**. Most cells migrated into the retinal layer when Chondroitinase ABC was used (**H**, **P**). The cells express GFAP whether in the subretinal space (**L**) or vitreous cavity (**P**). None of the cells located in the retinal layers express GFAP. Scale bar: 20 μm (**D**, **H**, **L**, **P**) Abbreviations: ganglion cell layer (GCL); inner plexiform layer (IPL); inner nuclear layer (INL); outer nuclear layer (ONL); and subretinal space (SRS).

We next transplanted mRPC into the *Rho*^−/−^ mice. Similarly, chondroitinase ABC downregulated the chondroitin expression around the outer nuclear area that was induced by transplantation (Figure 4D,H). The cells located in the subretinal space or vitreous cavity expressed GFAP, whether in the control or chondroitinase ABC group (Figures 4L,P). The GFAP expression in retinal layers in the chondroitinase ABC group was the same as in the control group. However, chondroitinase ABC promoted substantial migration of the cells into the retinal layers. GFAP expression was not seen around the donor cells that resided in the retinal layers (Figure 4P). Many cells migrated into the inner nuclear layer and inner plexiform layer with the employment of chondroitinase ABC (Figure 4Q).

**Figure 5:**
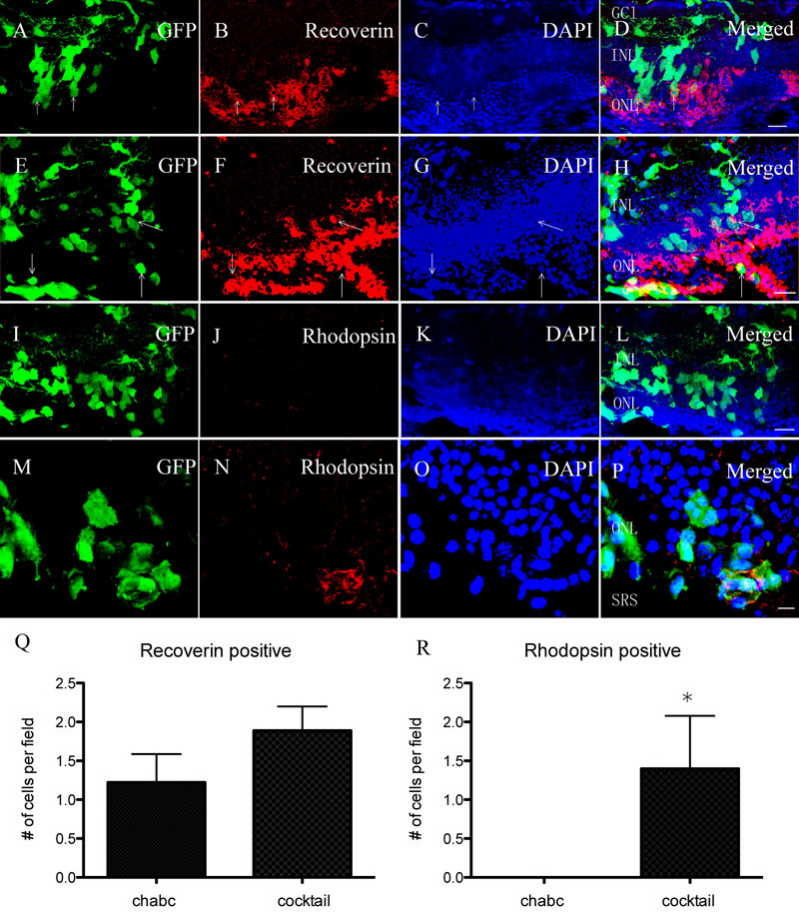
Transplantation of murine retinal progenior cells (mRPCs) into *Rho*^−/−^ mice combined with chondroitinase ABC or a cocktail. The first row (**A**-**D**) and the third row (**I**-**L**): Transplantation with chondroitinase ABC. **Th**e second row (**E-H**) and the fourth row (**M-P**): Transplantation with a cocktail. The average number of recoverin-positive (**Q**) or rhodopsin-positive cells (**R**) was counted per microscopic field. The columns are mean values, error bars are SD *p<0.05 (n=9). The cells (arrow) are recoverin-positive in the outer nuclear layers and inner nuclear layers (**D**, **H**). The cells express rhodopsin when they are transplanted with a cocktail (**N**). However, no rhodopsin-positive cells are seen in **J**. Scale bar: 20 μm (**D**, **H**, **L**); 10 μm (**P**). Abbreviations: ganglion cell layer (GCL); inner nuclear layer (INL); outer nuclear layer (ONL); and subretinal space (SRS).

**Figure 6:**
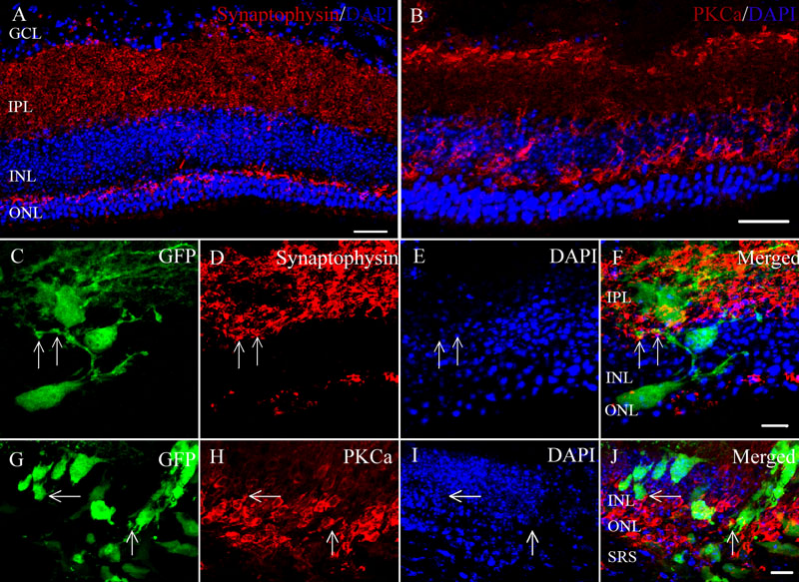
Integration of murine retinal progenior cells (mRPCs) after transplantation with a cocktail (Chondroitinase ABC 0.01 U/ml, DAPT 10 uM, IGF1 10 ng/ ml) into *Rho*^−/−^ mice. **A**: The untreated control group (Synaptophysin). **B**: The untreated control group (protein kinase alpha-PKCα). The second row (**C**, **D**, **E**, **F**) and the third row (**G**, **H**, **I**, **J**) were cocktail treated group. The right panel is a merged image of the three panels to the left. Integrated cells express the synaptophysin (arrow in **D**), and appear to make synaptic contact with the bipolar cells (arrow in **H**). Scale bar: 20 um (**A**, **B**); 10 um (**F**, **J**). Abbreviations: ganglion cell layer (GCL); inner plexiform layer (IPL); inner nuclear layer (INL); and outer nuclear layer (ONL); and subretinal space (SRS).

To determine if the growth factors could induce differentiation in vivo, a cocktail (Chondroitinase ABC 0.01 U/μl, DAPT 10 μM, IGF1 10 ng/ml) was transplanted with mRPCs into the *Rho*^−/−^ mice. After transplantation with the cocktail, more cells expressed rhodoposin and integrated into the outer nuclear layer (Figure 5P,R). A cluster of cells were recoverin-positive and located in the outer nuclear layer (Figure 5D,H,Q). There was no significant difference in recoverin-positive cells between the control (Figure 5D) and cocktail group (Figure 5H). The untreated control group is shown in Figure 6A (Synaptophysin) and Figure 6B (PKCα). Normally, synaptophysin is expressed in the inner plexiform layer, whereas PKCα expression is located in the inner nuclear layer. The mRPCs extended their neurites to the inner plexiform layer, which is a key location for synaptic activity (Figure 6F). It appeared that donor cells extended their neurites in the host bipolar-cell layer in morphology (Figure 6J).

## Discussion

Cell-replacement therapy provides a promising method for treating irreversible retinal degenerative diseases. Choosing a cell source for transplantation is always controversial. Previously, retinal sheets were used for transplantation [13,35,36]. Unfortunately, intact embryonic retinal sheets did not interact or integrate with host neurons. Integration with the host retina is much better in the transplantation of retinal progenitor cells. These cells are derived from developing retina. They can be maintained in cell culture and will continue to proliferate and generate new neurons and specialized retinal cells. When these cells are transplanted into either the normal or degenerate (dystrophic) retinas of rats and mice, they can migrate into all retinal layers and develop morphological characteristics of various retinal cell types [37,38]. Though recent studies show that Nrl+ postmitotic photoreceptor precursors and photoreceptors derived from human embryonic stem cells can integrate into the host retina and restore some visual function, mRPCs remain a viable cell source for the transplantation due to their easy accessibility and multipotency. Also, we found that RPCs were able to produce TGF-2, FasL, and IDO, indicating that RPCs could not only suppress immunity to the transplanted allogeneic progenitor cells, but could also create a local immune privilege site [39]. In addition, subretinal space is an immune privilege area. This cell- or tissue-specific immune privilege allows donor material to survive transplantation to an allogeneic host [40].

CSPGs play a vital role in the prevention of functional repair in the CNS. Since chondroitinase ABC has been successfully used to treat a spinal cord injury [41], several studies have focused on using chondroitinase to promote the functional recovery of CNS diseases [30,42-49]. Considerably less attention has been paid to the effects of chondroitinase ABC on eye diseases. So far, chondroitinase ABC shows promising effects for promoting the integration of grafted cells [22,27,28]. Our study shows that chondroitinase ABC improves the migration distance of donor cells in the retinal layers of B6 mice. The donor cells are surrounded by chondroitin, which is the main obstacle to their migration [22]. Removal of inhibitors promotes donor cell movement into the retinal layers. Unfortunately, most cells were in the inner nuclear layer and only a few cells expressed recoverin (a marker for rod and cone photoreceptors and some cone bipolar cells) and β-3-tubulin (data not shown). Issues such as these may be accounted for by a variety of factors, including overcrowding of the outer nuclear layer, the effects of chemotactic factors, and the lack of a permissive environment for differentiation. We hypothesize that an appropriate animal model will contribute to the migration and differentiation of grafted cells.

*Rho*^−/−^ mice are B6 background mice developed by targeted disruption of the rhodopsin gene, which is an animal model for retinitis pigmentosa. Due to the loss of rod outer segments and photoreceptors, the *Rho*^−/−^ mice may provide a permissive environment for migration and differentiation of donor cells. In our study, the GFAP expression was even stronger in the outer nuclear layer and in the vicinity of the donor cells in the subretinal space. Some recoverin-positive cells were located in the inner nuclear layer or inner plexiform layer. This finding may be caused by three major transformations induced by retinal degeneration: 1) Müller cell hypertrophy and elaboration of a distal glial seal between the retina and the choroid/retinal pigmented epithelium; 2) apparent neuronal migration along glial surfaces to ectopic sites; and 3) rewiring through evolution of complex neurite fascicles, new synaptic foci in the remnant inner nuclear layer, and new connections throughout the retina [50]. In the laminar-laminar retina culture experiment, accumulation of CRALBP, CD44, and neurocan were observed in the interface of the abutting retina pieces. Accordingly, such integration occurred exclusively where CRALBP, CD44, and neurocan immunolabeling appeared to be disrupted in the interface, but coincided with high GFAP expression within the rd1 retina. This implies that hypertrophy and GFAP expression are not sufficient or even appropriate indicators of a nonpermissive environment [14]. Chondroitinase ABC can disrupt the glial barrier and promote the migration of the donor cells. Once we were able to make the mRPCs arrive at the correct lamina, the differentiation became important for the ability to restore vision. We actually found that some cells resided in the outer nuclear layer and expressed recoverin. However, none of the donor cells differentiated into rhodopsin positive cells in a *Rho*^−/−^ host. This may be related to the complete absence of rhodopsin expression in *Rho*^−/−^ mice and a lack of incentive factors for rod differentiation. We hypothesize that the addition of known photoreceptor inducers may increase the levels of cellular differentiation.

In our study, the synergistic effects of chondroitinase, DAPT, and IGF-1 promoted the differentiation of donor progenitor cells into rhodopsin and recoverin positive cells. Simultaneously, donor progenitor cells had neuronal sprouting. It is puzzling that only a few cells differentiated into rod photoreceptors. There are some possible reasons for this. First, the *Rho*^−/−^ mice did not have the incentive environment for rod cell differentiation. Second, the growth factors might have degraded quickly after two days. If we had used the microsphere to release the growth factors slowly and continuously, it may have greatly contributed to photoreceptor development and induced more mRPCs to differentiate into rod photoreceptors. We also evaluated the visual function by ERG. However, we didn’t see the same results as in the previous study [6,7]. This may relate to a low subpopulation of photoreceptors, low rate of integration (<0.01%), or an inappropriate period for collecting the mRPCs. Studies show that the best ages for the donor cells are from P3 to P5, roughly corresponding to two or three days following peak rod photoreceptor production in a mouse retina [6]. The issue we have to address is how to capture enough photoreceptor precursors. However, in terms of translation into a clinical setting, it is not feasible to collect an abundance of rod or cone photoreceptor precursors from fetal retina for use in cell-replacement therapy. Therefore, the differentiation between retinal progenitor cells, embryonic stem cells, or induced pluripotent stem cells (iPSCs) is the best choice. It would be helpful to find the key genes or growth factors related to photoreceptor development. We have multiple options for this: 1) Infect the mRPCs with Crx or Nrl gene before transplantation. With development of molecular biology, new genes will be identified for the photoreceptor development. This approach will definitely promote the cell-based therapy. 2) Induce photoreceptor differentiation before transplantation. The complicated issue here is how long it will take to induce differentiation before transplantation. If the progenitor cells develop to mature photoreceptors they will lose their pluripotency and it will be difficult for them to migrate and integrate into retinal layers. 3) Perform transplantation with growth factors either directly or via a controlled release system.

In conclusion, our study shows that chondroitinase ABC and growth factors promote the migration and anatomic integration of mRPCs into Rho^−/−^ mice. In addition, it provides an innovative perspective for cell-based therapy.
